# Integrated Explainable Diagnosis of Gear Wear Faults Based on Dynamic Modeling and Data-Driven Representation

**DOI:** 10.3390/s25154805

**Published:** 2025-08-05

**Authors:** Zemin Zhao, Tianci Zhang, Kang Xu, Jinyuan Tang, Yudian Yang

**Affiliations:** 1 AECC Harbin Dongan Engine Co., Ltd., Harbin 150066, China; zeminzhao666@163.com; 2 State Key Laboratory of Precision Manufacturing for Extreme Service Performance, Central South University, Changsha 410083, China; 223712158@csu.edu.cn (K.X.); jytang@csu.edu.cn (J.T.); 3 College of Mechanical and Electrical Engineering, Central South University, Changsha 410083, China; 4 AECC Zhongchuan Transmission Machinery Co., Ltd., Changsha 410083, China; yangyudian202506@163.com

**Keywords:** gear wear, explainable fault diagnosis, deep learning, dynamic modeling

## Abstract

Gear wear degrades transmission performance, necessitating highly reliable fault diagnosis methods. To address the limitations of existing approaches—where dynamic models rely heavily on prior knowledge, while data-driven methods lack interpretability—this study proposes an integrated bidirectional verification framework combining dynamic modeling and deep learning for interpretable gear wear diagnosis. First, a dynamic gear wear model is established to quantitatively reveal wear-induced modulation effects on meshing stiffness and vibration responses. Then, a deep network incorporating Gradient-weighted Class Activation Mapping (Grad-CAM) enables visualized extraction of frequency-domain sensitive features. Bidirectional verification between the dynamic model and deep learning demonstrates enhanced meshing harmonics in wear faults, leading to a quantitative diagnostic index that achieves 0.9560 recognition accuracy for gear wear across four speed conditions, significantly outperforming comparative indicators. This research provides a novel approach for gear wear diagnosis that ensures both high accuracy and interpretability.

## 1. Introduction

Gears, as core components of mechanical transmission systems, directly influence the operational performance of transmission systems through their state of health. Under long-term alternating loads, gear tooth surfaces inevitably experience varying degrees of wear, leading to deterioration of meshing characteristics, reduced transmission efficiency, and increased vibration intensity. Severe wear may even cause abnormal equipment shutdowns and potential safety hazards [1]. Therefore, research on condition monitoring and diagnosis of gear tooth surface wear is crucial for ensuring the operational reliability of gear transmission systems.

Vibration analysis has become a mainstream technical approach for gear fault diagnosis due to its advantages of rich information dimensions, convenient signal acquisition, and high real-time performance [2,3]. Among these methods, dynamic model-based characterization of gear health conditions is particularly significant in gear fault diagnosis research, featuring strong interpretability and low dependency on historical data [4,5]. For instance, Liu et al. [6] proposed a vibration modeling and fault evolution symptom analysis method for gearbox diagnosis. Gao et al. [7] established a dynamic predictive model to analyze degradation through wear and cracking. Li et al. [8] presented a deterministic method of surface degradation to research the influence of rough topography evolution on dynamic responses. Zhang et al. [9] proposed a wear prediction method considering friction–wear coupling based on tribo-dynamic analysis. However, dynamics modeling-based gear health characterization methods rely heavily on prior knowledge, and their diagnostic accuracy is limited by model simplification assumptions with insufficient adaptability to measured data [10]. These limitations result in unsatisfactory fault diagnosis accuracy that fails to meet engineering requirements.

Vibration data-driven gear fault diagnosis methods have long been a key research focus in this field [11,12]. Traditionally, researchers have employed signal processing-based feature extraction techniques to analyze gear vibration data for fault identification [13,14,15]. For instance, Liu et al. [16] proposed a gear fault diagnosis method based on empirical mode decomposition and multi-fractal spectrum analysis. Hu et al. [17] developed a high-order synchrosqueezing wavelet transform method for gear vibration signal analysis. Teng et al. [18] utilized a novel vibration model combined with empirical wavelet transform for gear vibration analysis. However, these signal processing-based approaches require manual design of feature extraction rules and exhibit low data analysis efficiency [19,20], making them inadequate for meeting the real-time requirements of gear transmission system condition monitoring and fault diagnosis.

Recent advances in deep learning have provided powerful tools for rapid analysis of high-dimensional vibration data, making deep learning-based gear fault diagnosis a predominant research direction in this field [21,22,23]. For example, Dong et al. [24] developed a dynamic supervised contrastive learning network with multi-scale compound attention for gearbox fault diagnosis. Kang et al. [25] employed an Adam-optimized CNN-LSTM network for gearbox fault identification. Jia et al. [26] proposed a lifting wavelet-informed hierarchical domain adaptation network for accurate gearbox diagnosis. Guo et al. [27] introduced a convolutional attention fusion network for small-sample gearbox fault diagnosis. Zhuang et al. [28] designed a residual attention temporal recurrent network for fault identification with limited labeled data. However, while existing methods leverage deep learning’s adaptive fitting capability for complex data, they suffer from poor interpretability—the extracted fault features lack clear physical meaning [29,30]. Consequently, diagnostic results remain unintelligible to engineers, hindering their direct application in maintenance decision-making for industrial machinery.

**Figure 1:**
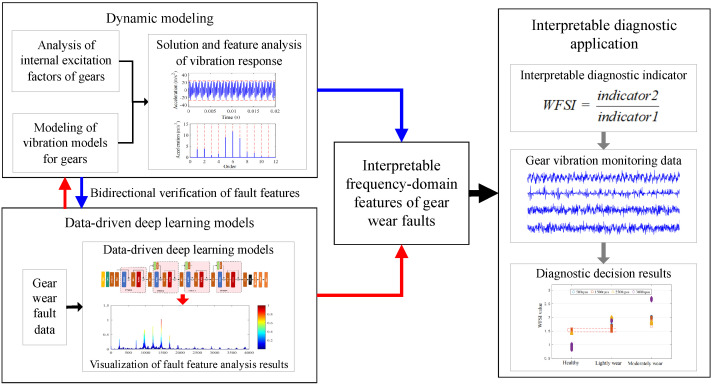
The overall structure of the proposed method.

To address these issues, this paper proposes a bidirectional verification framework that integrates dynamic modeling and data-driven representation for the frequency-domain characteristics of gear wear faults, achieving interpretable fault diagnosis, as shown in Figure 1. First, a dynamic model of gear tooth surface wear is established to reveal the modulation effects of different wear levels on time-varying meshing stiffness, transmission error, and vibration response spectra. Second, a deep convolutional neural network incorporating Gradient-weighted Class Activation Mapping (Grad-CAM) is designed to extract visualized frequency-domain sensitive features from measured vibration signals, enabling adaptive focusing on fault-sensitive frequency bands. Through bidirectional feature verification between the dynamic model and deep learning model, it is found that higher-order meshing frequencies are significantly enhanced in the vibration signals of gear wear faults, with a notable increase in meshing frequency energy ratio in envelope spectra. Based on this, an interpretable quantitative discriminant index for tooth surface wear faults is constructed, ultimately achieving accurate identification of gear wear faults. The proposed method overcomes the limitations of poor adaptability and weak interpretability in traditional approaches through bidirectional verification of dynamic mechanisms and deep learning representations, providing a new paradigm for gear fault diagnosis that combines high accuracy with interpretability. The specific contributions are as follows:

(1) A frequency-domain feature extraction method for gear wear faults is proposed, which combines bidirectional verification between dynamic modeling and data-driven representation, achieving physically constrained accurate characterization of gear wear states.
(2) An interpretable quantitative discriminant index for gear wear faults is established based on dynamic mechanisms and deep learning models, enabling accurate identification of gear wear faults under different wear severities.
(3) The effectiveness and superiority of the proposed method for interpretable gear fault diagnosis are verified through gear wear fault experiments.

The remaining sections are organized as follows: Section 2 presents the feature analysis of gear wear faults based on dynamic modeling. Section 3 describes the gear wear fault simulation experiments and the experimental data acquired. Section 4 details the data-driven visual analysis of fault features performed using deep learning. Section 5 constructs the gear wear fault discriminant index and describes fault identification experiments. Section 6 provides the conclusions of this study.

## 2. Feature Analysis of Gear Wear Based on Dynamic Modeling

In this section, we analyze the vibration characteristics of gear wear faults from a mechanistic perspective through dynamic modeling.

Gear wear causes geometric deviations between actual tooth surfaces and designed tooth surfaces, affecting internal excitation factors during gear system operation, such as meshing stiffness and no-load static transmission error. These changes consequently influence the dynamic characteristics of the gear system. Accordingly, we analyze the effects of tooth surface wear on meshing stiffness and transmission error, establish a dynamic model of gear transmission, and investigate the fault signal characteristics of worn gears through vibration response analysis.

### 2.1. Effect of Gear Wear on Meshing Stiffness

During gear meshing, there exist single-tooth contact zones and double-tooth contact zones. According to elastic mechanics, the time-varying meshing stiffness $k_{\mathrm{single}}$ of gear pairs in the single-tooth contact zone can be derived as follows:

(1) $$\begin{array}{c} \begin{array}{c} k_{\mathrm{single}}=1/\left(\frac{1}{k_{h}}+\frac{1}{k_{t}^{p}}+\frac{1}{k_{f}^{p}}+\frac{1}{k_{t}^{g}}+\frac{1}{k_{f}^{g}}\right)\\ k_{t}^{n}=1/\left(\frac{1}{k_{a}^{n}}+\frac{1}{k_{b}^{n}}+\frac{1}{k_{s}^{n}}\right),n=p,g\; \end{array} \end{array}$$

where the superscripts *p* and *g* represent the driving gear and driven gear, respectively. $k_{h}$, $k_{t}$, and $k_{f}$ denote the nonlinear Hertzian contact stiffness, tooth stiffness, and foundation stiffness, respectively. $k_{a}$, $k_{b}$, and $k_{s}$ represent the radial compression stiffness, bending stiffness, and shear stiffness, respectively.

The meshing stiffness of worn gear pairs in the single-tooth contact zone is primarily influenced by the effective cross-sectional area and effective moment of inertia. However, since the changes caused by wear depth are extremely minimal and negligible, our focus shifts to examining the stiffness variation and load distribution ratio in the double-tooth contact zone of worn gear pairs. The meshing stiffness and load distribution coefficient in the double-tooth contact zone can be expressed as follows:

(2) $$\begin{array}{c} \left\{\begin{array}{c} K=\frac{K_{1}+K_{2}}{1+\left(K_{1}E_{12}/F_{n}\right)}\;E_{12}>0\\ K=\frac{K_{1}+K_{2}}{1-\left(K_{2}E_{12}/F_{n}\right)}\;E_{12}<0 \end{array}\right. \end{array}$$

(3) $$\begin{array}{c} \left\{\begin{array}{c} LSF_{1}=\frac{K_{1}}{K_{1}+K_{2}}\left(1-\frac{K_{2}E_{12}}{F_{n}}\right)\\ LSF_{2}=\frac{K_{2}}{K_{1}+K_{2}}\left(1+\frac{K_{1}E_{12}}{F_{n}}\right)\; \end{array}\right. \end{array}$$

The time-varying meshing stiffness after tooth surface wear can be calculated by incorporating the wear amount at different meshing positions as tooth profile errors into the analytical formula.

**Figure 2:**
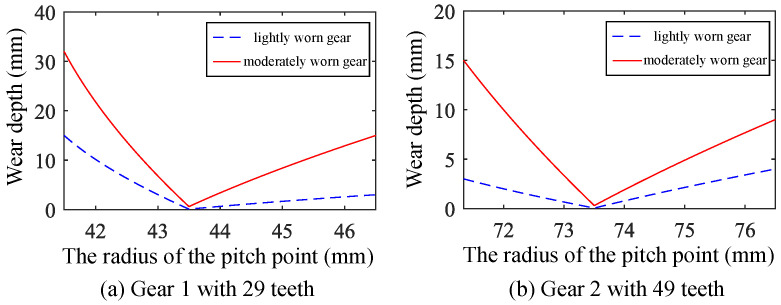
Wear depth of faulty gears.

For the healthy gear and two sets of fault gears with different wear levels, the time-varying meshing stiffness was calculated. The specific parameters of the healthy gear are listed in Table 1. The wear amount curves for the two sets of worn gears are shown in Figure 2.

**Figure 3:**
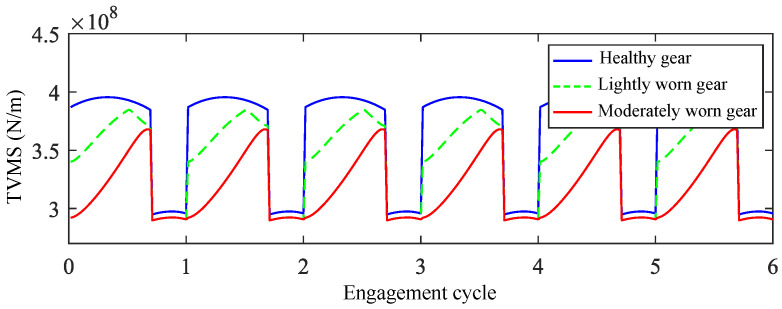
TVMS of healthy and differently worn gears.

Based on the basic gear parameters and wear depth data, the calculated time-varying meshing stiffness (TVMS) curves are shown in Figure 3. The results indicate that after tooth surface wear occurs, the meshing stiffness in the single-tooth zone decreases slightly, while in the double-tooth zone, the meshing stiffness shows a significant reduction, with the most pronounced decrease occurring at the transition position from the single-tooth to the double-tooth zone.

### 2.2. Effect of Gear Wear on No-Load Static Transmission Error

The no-load static transmission error refers to the deviation between the actual rotation angle and the ideal rotation angle of gears caused by tooth surface deviations from the ideal involute profile under no-external-load conditions. The no-load static transmission error consists of four components: long-period error $e_{L}$, short-period error $e_{S}$, random error $e_{R}$, and wear-induced error $e_{W}$. Its formula is expressed as follows:

(4) $$\begin{array}{c} e_{s}\left(t\right)=e_{L}+e_{S}+e_{R}+e_{W} \end{array}$$

(5) $$\begin{array}{c} \left\{\begin{array}{c} e_{L}=F_{i}^{\prime }\sin\left(2\pi f_{s}t\right)\\ e_{S}=f_{i}^{\prime }\sin\left(2\pi f_{m}t\right)\\ e_{W}=\min\left({\left[h_{wi}^{p}+h_{wi}^{g}\right]}_{i=1,2}\right)\\ e_{R}=0.2\mathrm{rand}\; \end{array}\right. \end{array}$$

where the tangential composite deviation amplitude of the tooth $f_{i}^{\prime }$ is taken as 15 μm; $f_{s}$ and $f_{m}$ represent the rotational frequency and meshing frequency, respectively.

**Figure 4:**
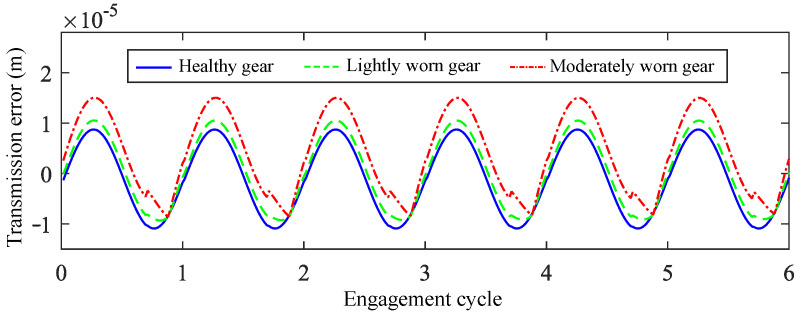
No-load static transmission error of healthy and worn gears.

By substituting the tooth surface wear depth results into Equations (4) and (5), the no-load static transmission error curves under different wear levels are obtained, as shown in Figure 4. It can be observed that as wear severity increases, the amplitude of no-load static transmission error grows, with sudden amplitude changes occurring at certain positions.

### 2.3. Vibration Characteristic Analysis of Gear Wear Based on Dynamic Model

To establish the dynamic model of gear pairs, the spatial beam element theory is adopted to model elastic support shafts. The spur gear-rotor system is equivalently represented as shaft elements with coupled interactions, where gear bodies are simplified as rigid rotors rotating with the shafts. The meshing effects of gear pairs are simulated by considering the time-varying meshing stiffness, static transmission error, and tooth side clearance, thereby establishing a multi-shaft split-torsion gear-rotor system dynamic model.

The gear-rotor system includes gears, elastic shaft segments, bearings, and input/output loads. Based on the finite element method’s meshing concept, the input and output shafts are discretized. The principles for dividing shaft nodes are as follows:

(1) Nodes are created at shaft endpoints and cross-section mutation points (shaft segment nodes).
(2) Nodes are placed at bearing support locations (support nodes).
(3) Nodes are assigned at gear meshing positions (meshing nodes).
(4) Nodes are established at load application points (load nodes).

**Figure 5:**
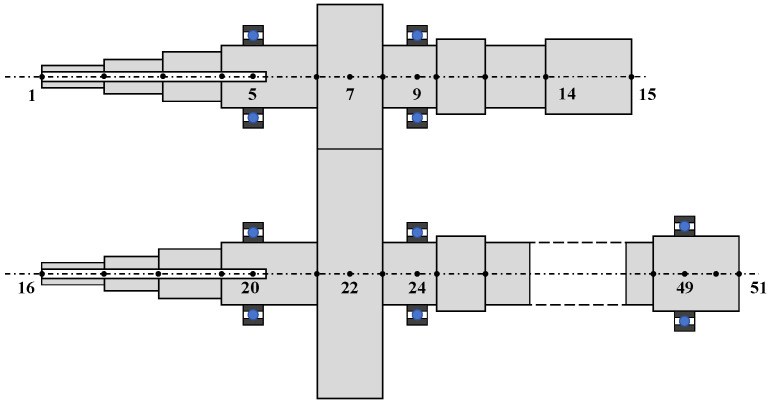
Finite element model of gear-rotor system.

The finite element model of the analyzed gear-rotor system is shown in Figure 5. The input and output shafts are divided into 15 and 36 finite element nodes, respectively. The driving gear rotor is located at Node 7, while the driven gear rotor is positioned at Node 22. Bearings are installed at Nodes 5, 9, 20, 24, and 49. The gear pair parameters are listed in Table 1, and the elastic shaft parameters are provided in Table 2.

**Figure 6:**
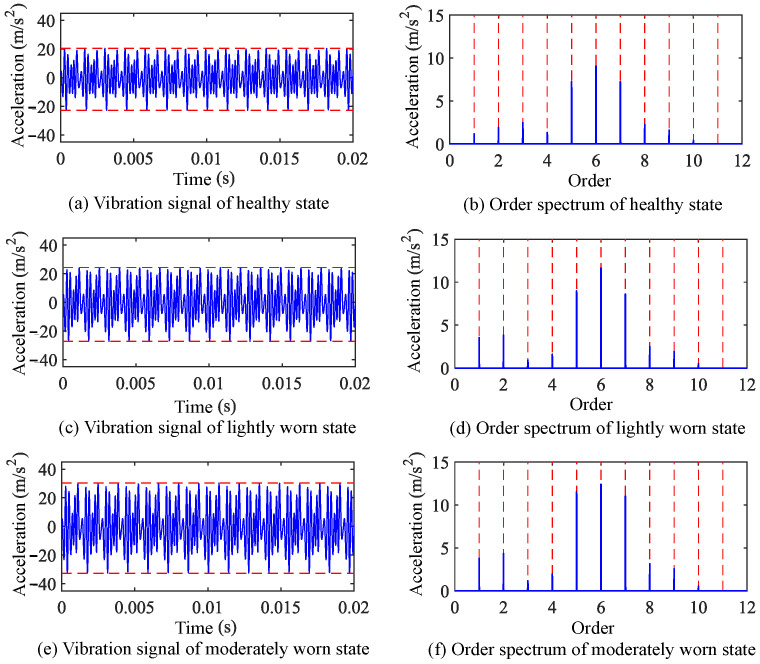
Time-domain waveform and order spectrum of vibration response signals under 1500 rpm operating conditions.

**Figure 7:**
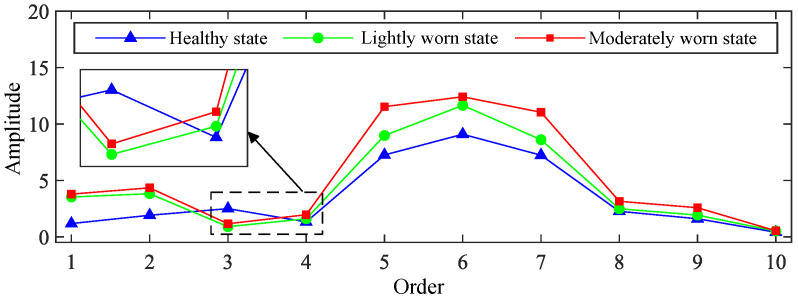
Amplitude values of meshing frequency and its harmonics in vibration acceleration signals under 1500 rpm operating conditions.

The time-varying meshing stiffness and no-load static transmission error of both healthy and worn gears, as calculated previously, were incorporated into the established finite element model of the gear-rotor system. The Newmark method was employed to solve the system’s dynamic response, yielding vibration response signals. Taking the 1500 rpm operating condition as an example, the vibration signal responses and their frequency-domain information for healthy and worn states are shown in Figure 6 and Figure 7.

The following can be observed after tooth surface wear occurs:

(1) The peak-to-peak value, root mean square value, and kurtosis index of vibration acceleration response all increase.
(2) No new characteristic frequencies or distribution patterns emerge in the response.
(3) The primary changes manifest as a growth in the amplitude of the meshing frequency and its harmonics.
(4) The growth of higher-order meshing frequency components is more pronounced compared to that of lower-order ones.

The dynamic model analysis results demonstrate that gear wear faults can cause significant increases in higher-order meshing frequencies within vibration response signals. These fault characteristics are derived entirely from dynamic model analysis, thus possessing strong interpretability.

## 3. Gear Wear Fault Simulation Experiment

In this section, we describe how we conducted simulation experiments of gear wear faults to obtain corresponding fault signals and perform preliminary analysis to further explore the vibration characteristics of gear wear faults. Additionally, the experimental fault signals acquired in this stage were used for the subsequent construction of data-driven deep neural networks and the final application of interpretable fault diagnosis.

### 3.1. Introduction to Gear Wear Experiment

**Figure 8:**
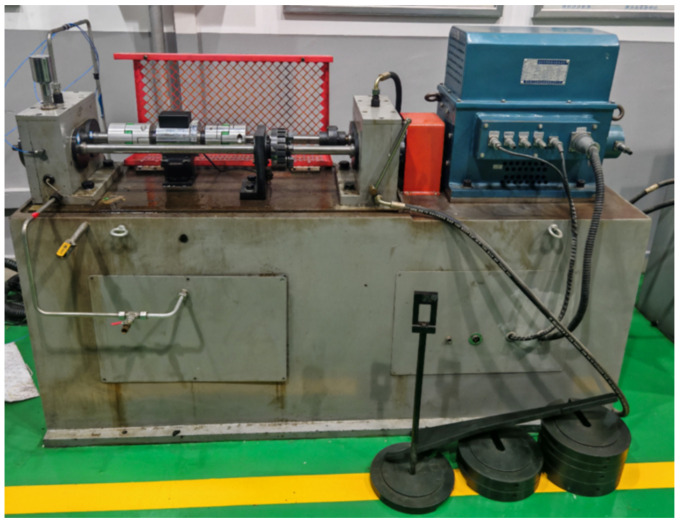
Gear wear fault test rig.

In this stage, we processed the tooth surfaces of healthy gears to simulate tooth surface wear faults. Experiments were conducted using both healthy gears and worn gears, with the vibration signals of the gearbox collected under various operating conditions. The experimental setup mainly consisted of a drive motor, test gearbox, companion gearbox, torque loading device, and high-precision measurement system, as shown in Figure 8.

**Figure 9:**
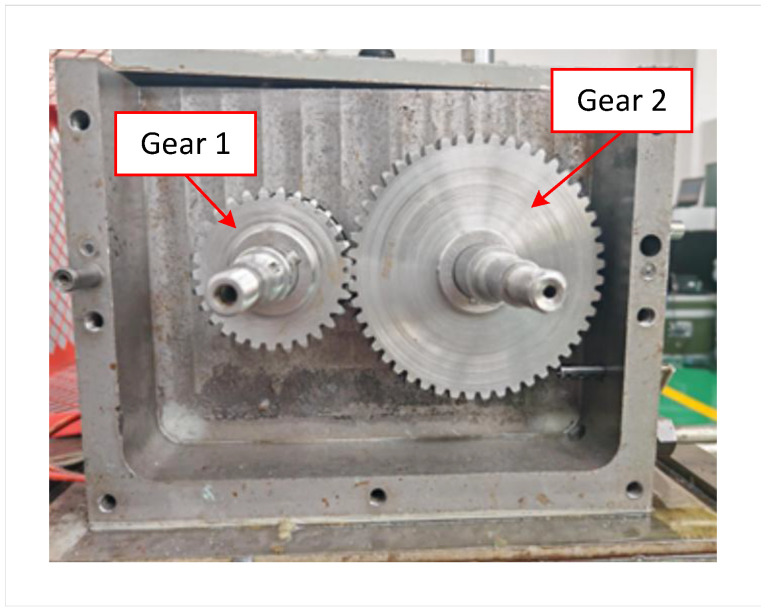
Gear assembly conditions.

The basic parameters of the adopted gear pair are shown in Table 1. The tooth profile of the worn gear was machined according to the wear depth shown in Figure 2, resulting in three sets of test gears: one set of healthy gears, one set of lightly worn gears, and one set of moderately worn gears. The gear assembly conditions are shown in Figure 9.

**Figure 10:**
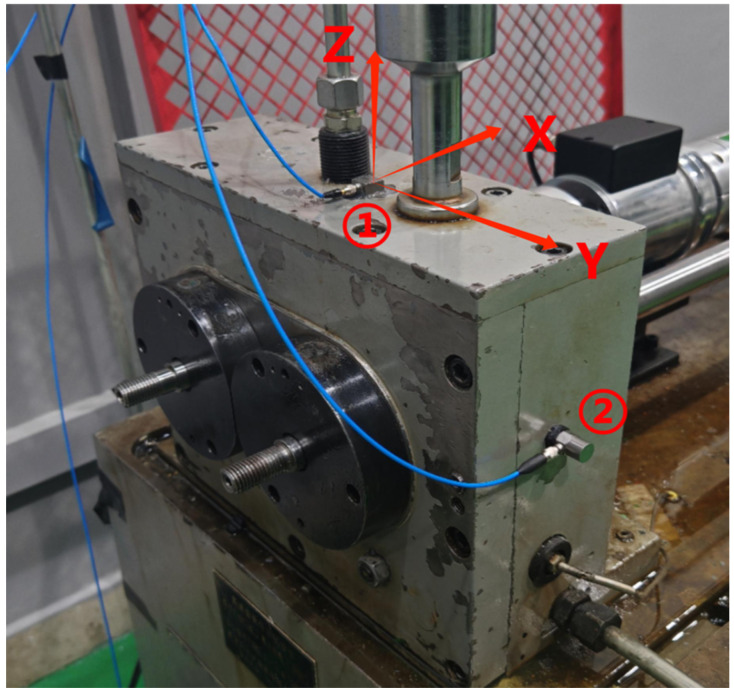
The sensor installation in the experiment. The circled numbers 1 and 2 in the figure denote two vibration sensors.

A PCB-353B18 accelerometer (PCB Piezotronics GmbH, Buffalo, United States) with a frequency response range of 0.7–30,000 Hz was installed on the gearbox housing to collect vibration signals. The measurement point was located near the gear shaft support bearing. The sensor installation is shown in Figure 10. The experimental operating conditions are listed in Table 3. For each test condition, the signal acquisition duration was 60 s, with a sampling frequency of 51.2 kHz.

### 3.2. Analysis of Gear Wear Vibration Signals

**Figure 11:**
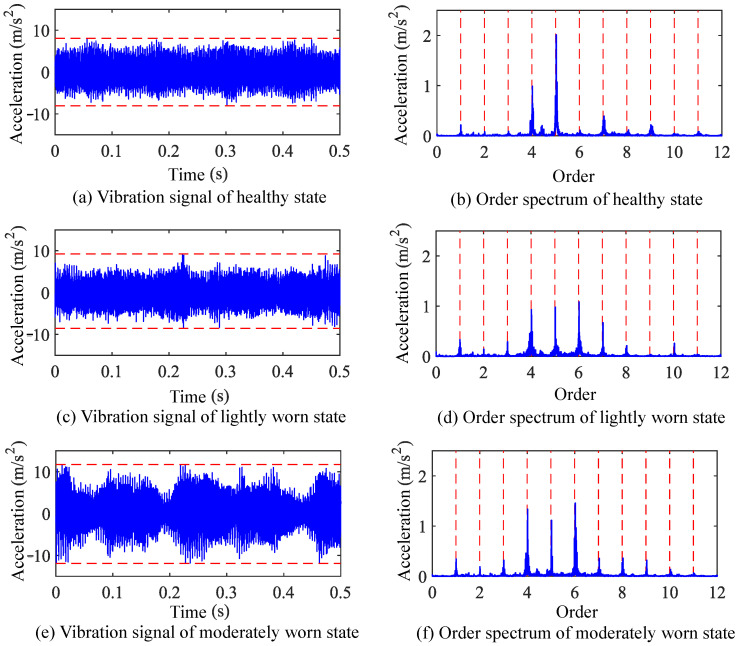
Gear vibration signals and order spectra under 500 rpm operating condition.

Taking the vibration signals under 500 rpm operating conditions as an example, the waveform and order spectrum of these signals are shown in Figure 11. Comparing the time-domain signals, for healthy, lightly worn, and moderately worn states, the peak-to-peak values are 35.8 m/s^2^, 40.2 m/s^2^, and 55.4 m/s^2^, respectively. The RMS values are 2.62 m/s^2^, 6.81 m/s^2^, and 7.67 m/s^2^, respectively. The kurtosis values are 2.46, 2.76, and 2.89, respectively. Compared with the healthy state, the worn states show increases in the peak-to-peak value, RMS value, and kurtosis.

For the order spectra, within the first ten meshing frequency orders, no significant new characteristic frequencies are observed in the lightly or moderately worn states compared to the healthy state. The main changes in the vibration signal order spectra after gear wear are concentrated in the amplitudes of meshing frequency and its harmonics. These observations are essentially consistent with the dynamic analysis results.

**Figure 12:**
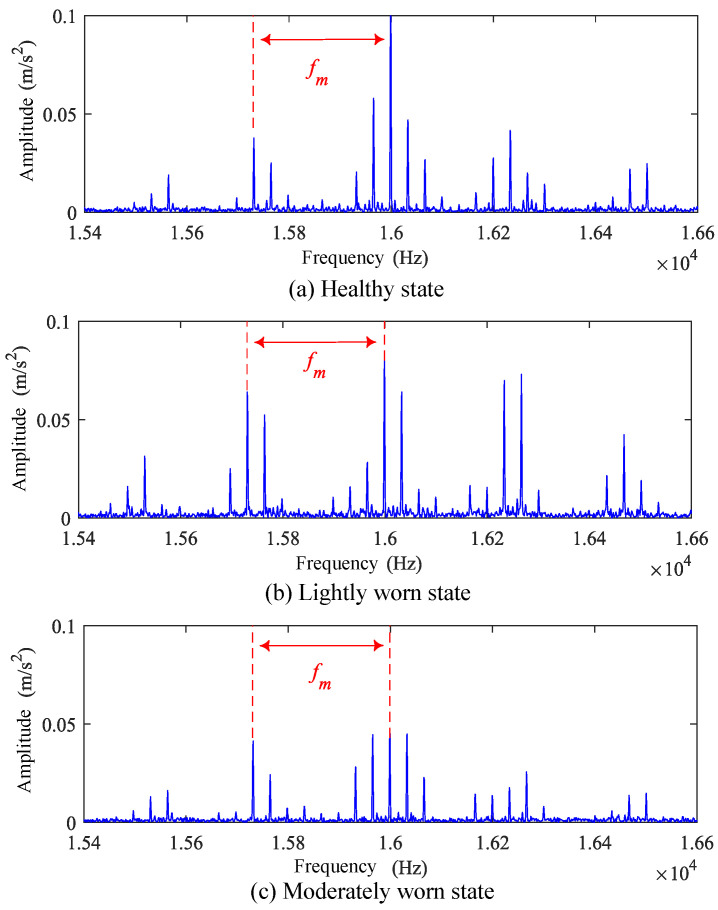
High-frequency band spectra of test gear vibration signals under 500 rpm operating conditions.

Additionally, the envelope spectrum of the vibration signals was plotted. It can be observed that in the high-frequency range of these signals, modulation phenomena with meshing frequency as the modulation spectrum are detectable, as shown in Figure 12.

In the high-frequency component envelope spectra of the healthy gear, lightly worn gear, and moderately worn gear signals, the first-order meshing frequency amplitudes are 0.16, 0.15, and 0.20, respectively. The second-order amplitudes are 0.09, 0.04, and 0.08, respectively. The third-order amplitudes are 0.04, 0.02, and 0.06, respectively. Therefore, in the high-frequency envelope spectra of wear fault signals, the energy ratio of the meshing frequency and its harmonic components to the total energy increases significantly.

## 4. Data-Driven Analysis of Sensitive Features for Gear Wear Faults

In this section, we describe how we employed a deep residual convolutional neural network to analyze the signal characteristics of gear wear faults from a data-driven perspective, and compare the analysis results with the previously mentioned dynamic modeling results for mutual verification.

### 4.1. Data-Driven Model Based on Residual Convolutional Network and Grad-CAM

The computational process of the constructed deep residual convolutional network mainly includes convolutional layers, pooling layers, and fully connected layers. Among these, the convolutional layer serves as the core component of the CNN, extracting local features through sliding operations of convolution kernels on input data. For a one-dimensional input sequence $X\in {ℜ}^{L}$, the mathematical representation of the convolution operation can be expressed as follows:

(6) $$\begin{array}{c} S\left(i\right)=\sum_{k=1}^{m}X\left(i+k-1\right)\cdot W\left(k\right)+b\; \end{array}$$

where $W\in {ℜ}^{m}$ represents the convolution kernel (with *m* being the kernel size), *b* is the bias term, and S(i) denotes the *i*th element of the output feature map. The stride parameter *s* controls the sampling interval of the feature map, while the padding parameter *p* determines the boundary processing method.

The pooling layer is typically connected after the convolutional layer and activation function. Common pooling operations include max pooling and average pooling. The fully connected layer serves as the key module in convolutional neural networks for classification decision-making. It maps the features extracted by convolutional layers to the target category space through global feature integration. Mathematically, the fully connected layer performs linear transformation and nonlinear mapping on the input feature vector $x\in {ℜ}^{D}$, generating category confidence scores:

(7) $$\begin{array}{c} z=\mathrm{Wx}+b,\;p=\mathrm{Softmax}(z)\; \end{array}$$

where $W\in {ℜ}^{M\times D}$ is the weight matrix, $b\in {ℜ}^{M}$ is the bias vector, *M* is the number of categories, and $z\in {ℜ}^{M}$ is the un-normalized category score vector. The Softmax function converts these scores into a probability distribution $p\in {ℜ}^{M}$ through exponential normalization, with the probability for class *c* given by

(8) $$\begin{array}{c} p_{c}=e^{z_{c}}/\sum_{k=1}^{M}e^{z_{k}} \end{array}$$

Based on the ResNet architecture, a one-dimensional convolutional neural network was constructed. The main structure of this network consists of one convolutional layer, four residual blocks, one fully connected layer, and one Softmax classification layer. Furthermore, each residual block contains three convolutional layers and three batch normalization layers. The built residual convolutional network enables data-driven adaptive extraction of gear wear fault features.

**Figure 13:**
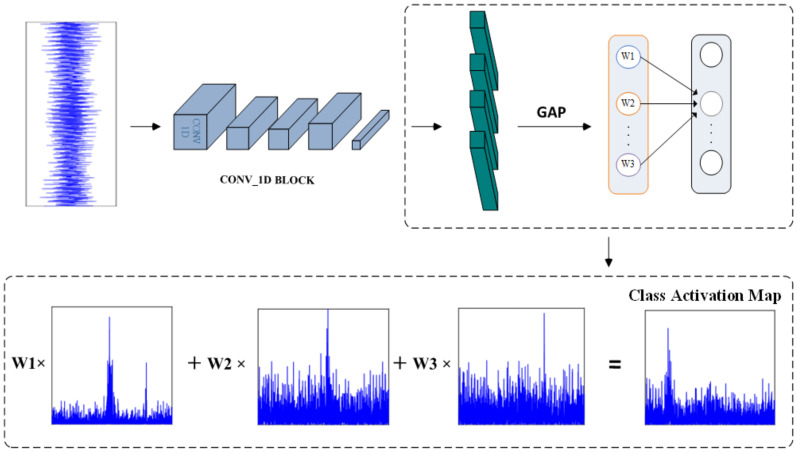
Schematic diagram of CAM acquisition process.

The deep residual convolutional network constructed above can achieve adaptive feature extraction for gear wear faults, but cannot visualize the extracted features. To highlight the frequency-domain-sensitive features of gear wear, and thereby help us to design reliable diagnostic indicators, we employed feature visualization techniques to visualize the features learned by the deep model. Class Activation Mapping (CAM) is a standard tool for visualizing features learned by convolutional neural networks. It can accurately locate the regions in the data features that are most relevant to the prediction results, as shown in Figure 13. Compared to other model interpretability and visualization methods, CAM is more compatible with the structure of the constructed deep residual convolutional network and offers higher computational efficiency, making it better suited to our needs.

For a one-dimensional CNN, let the feature maps output by the final convolutional layer be denoted as $A\in {ℜ}^{K\times L}$, where *K* represents the number of feature maps and *L* is the sequence length. Assuming the network uses global average pooling (GAP) followed by a fully connected layer for classification, spatial compression is performed on each feature map through global average pooling:

(9) $$\begin{array}{c} F_{k}=\frac{1}{L}\sum_{i=1}^{L}A_{k}\left(i\right),\;k=1,2,\ldots ,K \end{array}$$

The fully connected layer’s weight matrix $W\in {ℜ}^{C\times K}$ (where *C* is the number of categories) maps the compressed features to the category score space. For target category *c*, its prediction score $S_{c}$ can be expressed as

(10) $$\begin{array}{c} S_{c}=\sum_{k=1}^{K}w_{c}^{\left(k\right)}F_{k}=\frac{1}{L}\sum_{k=1}^{K}w_{c}^{\left(k\right)}\sum_{i=1}^{L}A_{k}\left(i\right) \end{array}$$

The class activation map $M_{\mathrm{CAM}}^{c}\in {ℜ}^{L}$ is generated by weighted summation of feature maps:

(11) $$\begin{array}{c} M_{\mathrm{CAM}}^{c}\left(i\right)=\sum_{k=1}^{K}w_{c}^{\left(k\right)}A_{k}\left(i\right),\;i=1,2,\ldots ,L \end{array}$$

Each element value in $M_{\mathrm{CAM}}^{c}\left(i\right)$ reflects the contribution degree of the corresponding position element in the sample to the classification decision of category *c*, with higher values indicating greater contribution.

In practical applications, the above method for obtaining CAM requires the network architecture to include a GAP layer. For networks that don’t meet this condition, the network structure needs modification and model retraining, making it inconvenient for direct application to other complex models. Additionally, the resolution of the final class activation map is limited by the size of the final convolutional feature map.

Gradient-weighted Class Activation Mapping (Grad-CAM) is an extension of Class Activation Mapping (CAM). By incorporating gradient information, it eliminates CAM’s dependency on network architecture, and can be applied to convolutional neural networks of any structure. Its core concept involves using the gradient of target class scores with respect to convolutional feature maps as weights to generate heatmaps that are spatially aligned with the input samples, thereby locating key regions relied upon by the model for decision-making. For one-dimensional deep convolutional neural networks, the specific steps to obtain CAM sequences are as follows:

(1) Load the trained model, input the spectrum sequence sample, and perform forward propagation to obtain class scores $S_{c}$.
(2) Fix network parameters, perform backpropagation to calculate the gradient of class scores $S_{c}$ with respect to the output feature maps $A\in {ℜ}^{K\times L}$ of the last convolutional layer, and use the global average of gradients as weights $\alpha_{k}^{c}$ for each channel.
  (12) $$\begin{array}{c} \alpha_{k}^{c}=\frac{1}{L}\sum_{i=1}^{L}\frac{\partial S_{c}}{\partial A_{k}\left(i\right)} \end{array}$$
(3) Perform weighted summation of channel weights and feature maps, then apply the ReLU activation function to retain positively correlated regions, obtaining the CAM sequence $M_{c}^{\mathrm{Grad}-\mathrm{CAM}}$.
  (13) $$\begin{array}{c} M_{\mathrm{Grad}-\mathrm{CAM}}^{c}\left(i\right)=\mathrm{ReLU}\left(\sum_{k=1}^{K}\alpha_{k}^{c}A_{k}\left(i\right)\right) \end{array}$$
(4) Interpolate and upsample the CAM sequence to restore it to the input sample size.
  (14) $$\begin{array}{c} M_{\mathrm{upsampled}}^{c}=\mathrm{Interpolate}\left(M_{\mathrm{Grad}-\mathrm{CAM}}^{c},\mathrm{mode}=‘\mathrm{linear}’\;\right) \end{array}$$

Through this process, the adaptive features learned by the residual convolutional network can be visualized and further used to identify fault-sensitive features of gear wear.

### 4.2. Data-Driven Model Training and Testing for Gear Wear Faults

The experimental gear wear fault data was used to train the constructed data-driven model. For the total duration *T* of data under each operating condition, the samples were divided according to the following rules. The starting time point of each sample is $t_{i}^{begin}\left(i=1,2,3,\ldots n\right)$, and the ending time point is $t_{i}^{end}\left(i=1,2,3,\ldots n\right)$, where $t_{i}^{begin}$, $t_{i}^{end}$, and *i* satisfy the following conditions:

(15) $$\begin{array}{c} \left\{\begin{array}{c} i=floor(\frac{T}{t_{const}-\Delta t})\\ t_{i}^{end}-t_{i}^{begin}=t_{const}\\ t_{i+1}^{begin}-t_{i}^{begin}=\Delta t \end{array}\right. \end{array}$$

For the experimental data in this study, the sample division parameters were set as $t_{const}$ = 1 s and Δt = 0.5 s. After data division, each speed condition under healthy, lightly worn, and severely worn states contained 2400 samples. All one-dimensional vibration signals in the samples were transformed into frequency-domain data sequences using the Discrete Fourier Transform (DFT). The final input to the model was single-channel sequence data with a length of 25,600. The DFT calculation formula is as follows:

(16) $$\begin{array}{c} X\left[k\right]=\sum_{n=0}^{N-1}x\left[n\right]\cdot e^{-j\frac{2\pi }{N}kn},\;k=0,1,\ldots ,N-1 \end{array}$$

where x[n] represents the time-domain data sequence; X[k] represents the frequency-domain complex sequence; and the exponential term $e^{-j\theta }$ corresponds to Euler’s formula cosθ−jsinθ.

**Figure 14:**
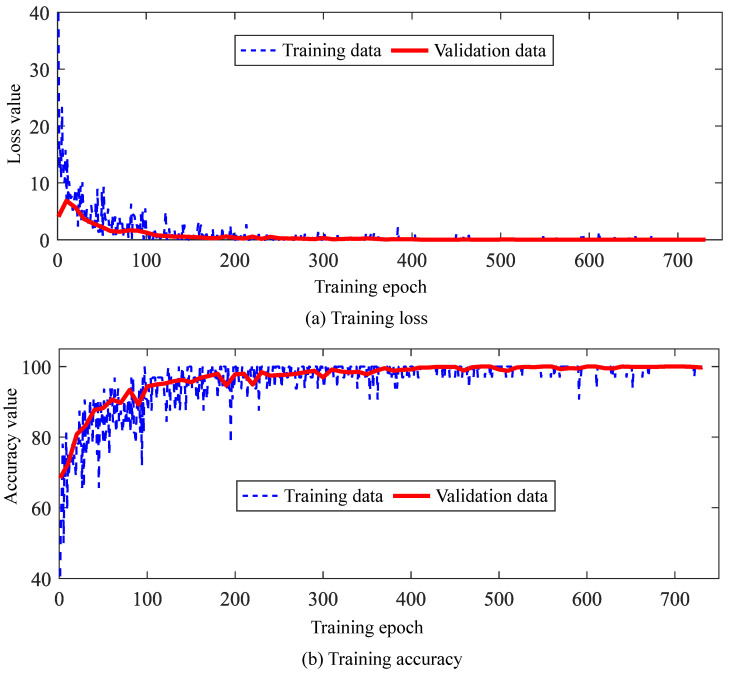
The accuracy and loss values during the training process.

All experimental samples under each operating condition were randomly shuffled, with 70% selected as the training set, 10% as the validation set, and 20% as the test set. During training, the accuracy curves for the training and validation sets are shown in Figure 14a, while the loss function curves are shown in Figure 14b. After training completion, the test set data was input for prediction, achieving classification accuracies of 95.9% for healthy samples and 99.5% for fault samples. This demonstrates that the trained model has good diagnostic performance and effectively models the characteristics of fault data.

### 4.3. Visualization Analysis of Sensitive Features for Gear Wear Faults

**Figure 15:**
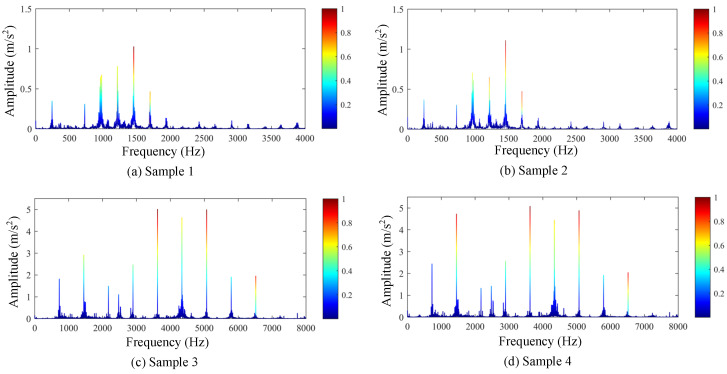
Superimposed results of gear wear state signal samples and CAM sequences.

After training the proposed data-driven model, four random wear state data samples were selected. The spectrum sequences and CAM sequences of these samples were superimposed through color mapping, resulting in the data curves shown in Figure 15.

The visualization results indicate that the constructed deep learning model primarily focuses on frequency regions below 10× meshing frequency when identifying gear wear conditions, with particular emphasis on the range between 4× and 8× meshing frequency. This suggests that variations within the 4× to 8× meshing frequency range contribute more significantly to characterizing gear wear. As shown in Figure 6 and Figure 7, the results from dynamic modeling demonstrate that the amplitudes of the 4× to 8× meshing frequencies increase after gear wear occurs. Therefore, the analysis results from the deep learning model are generally consistent with those from dynamic modeling, achieving interpretable and mutually validated identification of gear wear-related frequency-domain features.

## 5. Interpretable Feature Indicator Construction and Diagnostic Application for Gear Wear

In this section, we utilize the gear wear fault characteristics obtained through both dynamic modeling and data-driven representation to construct interpretable diagnostic indicators and apply them to gear wear fault diagnosis.

Specifically, the vibration signal characteristics after gear wear can be summarized as follows:

(1) The amplitudes of multiple meshing frequency orders increase, and the higher-order meshing frequency amplitudes show more significant growth.
(2) Modulation phenomena with meshing frequency as the modulation frequency become more prominent in high-frequency signal components, and the energy proportion of meshing frequency and its harmonics increases.

### 5.1. Diagnostic Indicator Design Based on Vibration Signal Characteristics

Based on these summarized characteristics, we design interpretable feature indicators for gear wear fault diagnosis. For a vibration signal data sample x(n) with 1 s duration, the detailed calculation process of the designed indicators is as follows:

(1) Calculate the spectrum of the original signal using the Discrete Fourier Transform:
  (17) $$\begin{array}{c} X\left(k\right)=\sum_{n=0}^{N-1}x\left(n\right)e^{-j2\pi kn/N},\;k=0,1,\ldots ,N-1 \end{array}$$
(2) Calculate the first 10 orders of meshing frequency for the current operating condition:
  (18) $$\begin{array}{c} \begin{array}{c} f_{m}=\frac{tacho}{60}\times z\\ f_{k}=k\cdot f_{m},\;k=1,2,\ldots ,10.\; \end{array} \end{array}$$
  where tacho is the driving shaft speed and *z* is the number of teeth on the driving gear.
(3) For each meshing frequency order $f_{k}$, search for the maximum amplitude in the frequency band range $\left[0.95f_{k},1.05f_{k}\right]$ within the spectrum sequence:
  (19) $$\begin{array}{c} A_{k}=\max\left(\left|X(f)\right|\right),\;f\in \left[0.95f_{k},1.05f_{k}\right] \end{array}$$
  Obtain the amplitude set $A=\{A_{1},A_{2},\ldots ,A_{10}\}$.
(4) Calculate the mean and standard deviation of set A:
  (20) $$\begin{array}{c} \begin{array}{c} \mu_{A}=\frac{1}{10}\sum_{k=1}^{10}A_{k}\\ \sigma_{A}=\sqrt{\frac{1}{9}\sum_{k=1}^{10}{\left(A_{k}-\mu_{A}\right)}^{2}} \end{array} \end{array}$$
  Define the ratio of mean to standard deviation as the factor indicator1:
  (21) $$\begin{array}{c} indicator1=\frac{\sigma_{A}}{\mu_{A}} \end{array}$$
(5) Filter the signal using a fourth-order Chebyshev IIR filter with a cutoff frequency of $f_{c}=10f_{m}$, obtaining the filtered signal:
  (22) $$\begin{array}{c} x_{\mathrm{hp}}\left(n\right)=\mathrm{II}R_{\mathrm{HPF}}\left[x(n)\right] \end{array}$$
(6) For the filtered signal, construct its analytic signal through the Hilbert transform:
  (23) $$\begin{array}{c} x_{\mathrm{analytic}}\left(n\right)=x_{\mathrm{hp}}\left(n\right)+j\cdot H\left\{x_{\mathrm{hp}}\left(n\right)\right\} \end{array}$$
  where H{·} represents the Hilbert transform. Then, extract the envelope signal and perform DFT on the envelope signal to obtain the envelope spectrum $X_{\mathrm{env}}\left(k\right)$ of the filtered signal:
  (24) $$\begin{array}{c} x_{\mathrm{env}}\left(n\right)=\left|x_{\mathrm{analytic}}\left(n\right)\right| \end{array}$$
  (25) $$\begin{array}{c} X_{\mathrm{env}}\left(k\right)=\sum_{n=0}^{N-1}x_{\mathrm{env}}\left(n\right)e^{-j2\pi kn/N} \end{array}$$
(7) Extract the first five orders of meshing frequency amplitudes from the envelope spectrum $X_{\mathrm{env}}\left(k\right)$:
  (26) $$\begin{array}{c} A_{k}^{\prime }=\max\left(|X_{\mathrm{env}}\left(f\right)|\right),\;f\in \left[0.97f_{k},1.03f_{k}\right],\;k=1,\ldots ,5. \end{array}$$
  Calculate the ratio of the first five orders’ meshing frequency energy to the total envelope spectrum energy, denoted as factor indicator2:
  (27) $$\begin{array}{c} indicator2=\frac{\sum_{k=1}^{5}{\left(A_{k}^{\prime }\right)}^{2}}{\sum_{k=0}^{N-1}|X_{\mathrm{env}}\left(k\right){|}^{2}} \end{array}$$
(8) The final designed Wear Fault Sensitivity Indicator (WFSI) is the ratio of indicator2 to indicator1:
  (28) $$\begin{array}{c} WFSI=\frac{indicator2}{indicator1} \end{array}$$

indicator2 reflects the intensity of meshing frequency modulation in high-frequency components. indicator1 reflects the stability of amplitude distribution across meshing frequency orders in the original signal and the smoothness of the multi-order meshing frequency amplitude curve. When gear wear occurs, the indicator2 value will increase, the indicator1 value will decrease, and the WFSI will increase.

### 5.2. Analysis of Gear Wear Fault Diagnosis Results

To verify the effectiveness of the constructed diagnostic indicator, the WFSI, we selected the root mean square (RMS) value, kurtosis value, and sum of meshing frequency order amplitudes of vibration signals (MFOA) as comparative diagnostic indicators.

In addition, two more advanced fault diagnosis indicators were selected for performance comparison. The first is the Degree of Cyclostationarity (DCS) [31], which is a cyclostationarity indicator of mechanical vibration signals. It measures the distance between the signal and its closest stationary process, and can be used for mechanical fault diagnosis. The second is the Envelope Spectrum Peak Factor (ESPF) [32], defined as the ratio of the maximum peak in the envelope spectrum to its root mean square value. It is particularly sensitive to early-stage mechanical faults. These indicators were then used to classify and identify the collected gear wear fault vibration signals.

**Figure 16:**
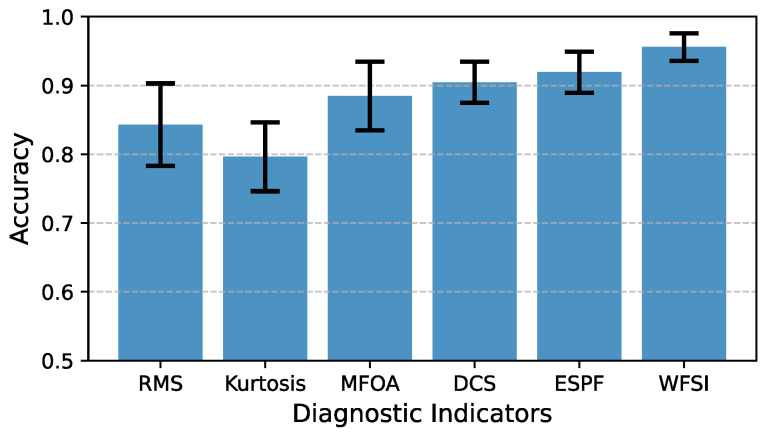
The accuracy results of fault diagnosis experiments.

**Figure 17:**
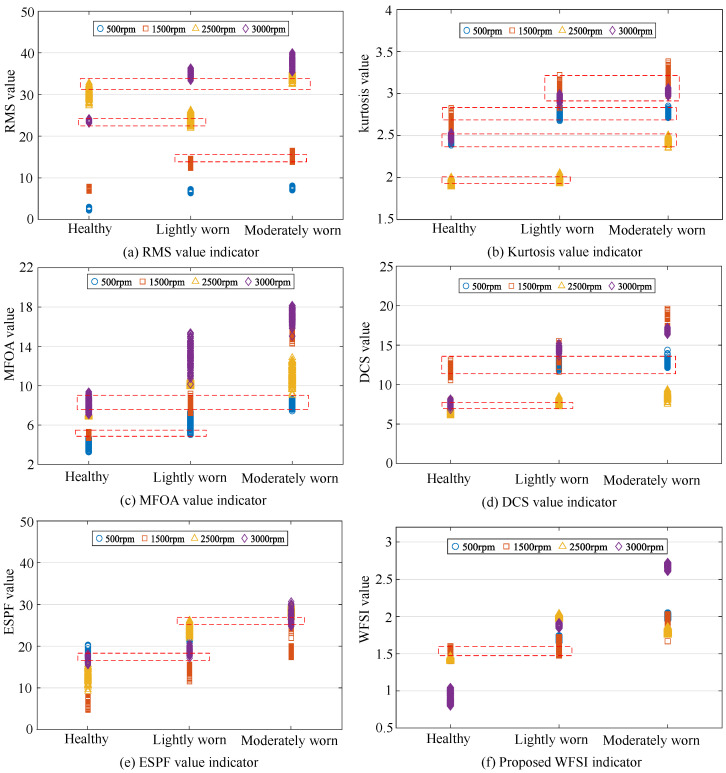
Diagnosis results of gear wear faults using different diagnostic indicators. The red dashed narrow boxes in each subfigure indicate misclassified samples.

Specifically, we randomly extracted 100 signal samples, each 1 s in length, from the vibration signals collected across all experimental groups. For each sample, we calculated the values of different diagnostic indicators and observed their distribution patterns. The fault diagnosis experiments were repeated 10 times, with signal samples randomly selected from the entire dataset in each trial, ensuring the reliability of the experimental results. The detailed results are presented in Figure 16 and Figure 17.

From a qualitative perspective, in the application results of the comparative diagnostic indicators (RMS, kurtosis, and MFOA), it can be observed that under the same rotational speed levels, the identification results of gear health conditions exhibit significant overlap and confusion. This implies that using these comparative diagnostic indicators for gear wear fault identification is prone to misdiagnosis. In contrast, for more advanced diagnostic indicators such as the DCS and ESPF, the overlap and confusion in diagnostic results are significantly reduced, although there is still a certain gap compared to the proposed diagnostic indicator, the WFSI. Specifically, the proposed interpretable diagnostic indicator, the WFSI, only shows confusion between healthy gears at 500 rpm and lightly worn gears at 1500 rpm, thereby enabling more effective identification of gear wear conditions.

From a quantitative perspective, the average diagnostic accuracy for the RMS indicator is 0.8430. For the kurtosis indicator, the average accuracy is 0.7963. For the MFOA indicator, the average accuracy is 0.8846. For the DCS indicator, the average accuracy is 0.9047. For the ESPF indicator, the average accuracy is 0.9193. For the proposed WFSI, the average accuracy reaches 0.9560. Based on the quantitative diagnostic results, the proposed interpretable diagnostic indicator demonstrates a clear advantage in accuracy over the comparative indicators. Therefore, this indicator combines both interpretability and high accuracy, providing a powerful tool for the precise identification of gear wear faults.

Finally, as shown in Equation (28), the proposed diagnostic indicator, the WFSI, is directly related to indicator1 and indicator2, making it necessary to conduct ablation experiments. Therefore, we carried out gear wear fault diagnosis experiments using indicator1, indicator2, and the proposed WFSI as diagnostic indicators, respectively. Each experiment was repeated ten times, and the average results were analyzed.

The results of the ablation experiments show that indicator1 achieved an accuracy of 0.8649 ± 0.03, while indicator2 achieved an accuracy of 0.8594 ± 0.02. In contrast, the proposed WFSI achieved the highest diagnostic accuracy (0.9560 ± 0.02). This demonstrates that the WFSI, constructed through Equation (28), has a stronger capability for identifying gear wear faults.

## 6. Conclusions

This paper proposes a bidirectional verification framework for the frequency-domain characteristics of gear wear faults that integrates dynamic modeling and data-driven representation. Furthermore, it constructs an interpretable diagnostic indicator for gear wear faults, thereby achieving highly accurate identification under the specified experimental conditions. The effectiveness of the proposed diagnostic indicator was validated using experimental gear wear fault data. The results show that the diagnostic indicator achieves an accuracy rate of 0.9560 for gear wear faults across four different speed levels, significantly outperforming the comparative indicators in this study. These results demonstrate the potential of the proposed diagnostic indicator as a promising approach for gear wear fault diagnosis, particularly due to its interpretability and high accuracy in the controlled laboratory environment.

For future research, first, factors such as tooth surface characteristics, friction, and lubrication after gear wear could be incorporated into dynamic modeling to obtain more precise gear wear fault features and enhance robustness. Second, critical for practical deployment, application studies on gear wear fault diagnosis must be conducted for non-stationary operating conditions with inherent industrial uncertainties, such as variable speed and variable load scenarios, to rigorously evaluate the method’s performance under realistic challenges.

## Figures and Tables

**Table 1:** Basic parameters of gears.

Parameter	Value
Module	3
Number of Teeth	29 (Gear 1), 49 (Gear 2)
Pressure Angle (°)	20
Face Width (mm)	20
Elastic Modulus (GPa)	206.8
Poisson’s Ratio	0.3

**Table 2:** Elastic shaft parameters.

Node Number	Outer Diameter (mm)	Inner Diameter (mm)	Length (mm)
1–2	16	6	34
2–3	20	6	23
3–4	24	6	20
4–6	30	6	30
6–10	30	0	70
10–11	34	0	25
11–13	35	0	42
13–15	38	0	45
15–17	16	6	34
17–18	20	6	23
18–19	24	6	20
19–21	30	6	30
21–25	30	0	70
25–26	40	0	25
26–48	28	0	500
48–51	30	0	63

**Table 3:** The experimental operating conditions.

Gear Condition	Test Speed (rpm)	Test Load (Nm)
Healthy	500	45
Light Wear	1500	
Moderate Wear	2500	
	3000	

## Data Availability

The gear wear experimental data used in this study is internal to the research team and is not publicly available. For more detailed data descriptions, the corresponding author of this paper can be contacted. Depending on the type of request, some of the important data will be provided appropriately.
